# PART and ARTAG tauopathies at a relatively young age as a concomitant finding in sporadic Creutzfeldt-Jakob disease

**DOI:** 10.1080/19336896.2021.1946378

**Published:** 2021-07-05

**Authors:** Kateřina Menšíková, Radoslav Matěj, Eva Parobková, Magdalena Smětáková, Petr Kaňovský

**Affiliations:** a Department of Neurology, University Hospital, Palacky University, Olomouc, Czech Republic; b Department of Neurology, Faculty of Medicine and Dentistry, Palacky University, Olomouc, Czech Republic; c Department of Pathology and Molecular Medicine, 3^rd^ Faculty of Medicine, Charles University and Thomayer Hospital, Prague, Czech Republic

**Keywords:** Sporadic Creutzfeldt-Jakob disease, age-related tauopathies, PART, ARTAG

## Abstract

Interactions between prion protein (PrP) and tau protein have long been discussed, especially in relation to the pathogenesis of neurodegenerative diseases. The presence of tauopathy in the genetic forms of Creutzfeldt-Jakob disease (CJD) brains is not uncommon. Molecular interactions between PrP and tau protein have been demonstrated in animal models; the role is attributed to the structural properties of misfolded isoform of the host-encoded prion protein (PrP^Sc^) aggregates, especially amyloid, which contributes to the phosphorylation of tau protein, which is reflected in the frequent occurrence of tau pathology in inherited prion amyloidoses. The question is the relationship between PrP^Sc^ and hippocampal tau pathology without amyloid deposits (i.e. PART and ARTAG) in sporadic CJD (sCJD). The co-occurrence of these two proteinopathies in sCJD brains is quite rare. These pathological entities have been described in only a few cases of sCJD, all of them were older than 70 years. There have been speculations about the possibility of accelerating the course of pre-existing tauopathy or the possibility of accelerating the ageing process in the CJD brains. Here we present the clinical course and neuropathological findings of a patient with sCJD in whom the above mentioned tauopathies PART and ARTAG, considered to be typical for older age, were found as early as 58 years of age. According to the available information, this case represents an unusually early occurrence of age-related tauopathies not only in relation to sCJD, but also in general.

## Introduction

Creutzfeldt–Jakob disease (CJD) is one subtype of the human prion diseases, a group of rapidly progressive lethal neurodegenerative diseases affecting humans and animals. They are associated with the accumulation of a misfolded isoform (PrP^Sc^) of the host-encoded prion protein (PrP). Human prion diseases are divided into sporadic, acquired and genetic forms. The sporadic forms, i.e. forms in which the source of infection could not be proven are sporadic CJD (sCJD), sporadic fatal insomnia (sFI) and a rare recently described unit called variably protease-sensitive prionopathy (VPSPr) [1]. The acquired forms are related to exposure to external prions and include kuru, iatrogenic CJD (iCJD) and variant CJD (vCJD). Genetic forms are associated with mutations in the *PRNP* gene and comprise genetic/familial CJD (gCJD), familial fatal insomnia (FFI) and PrP- amyloidoses, which include Gerstmann-Sträussler-Scheinker disease (GSS), PrP-cerebral amyloid angiopathy (PrP-CAA) and PrP systemic amyloidosis (PrP-SA) [2,3]. All listed clinical units differ in clinical picture, histopathological findings and rate of disease progression. In general, CJD and FI are rapidly progressive neurological syndromes, while the inherited PrP-amyloidoses manifest a slower clinical course and are mainly characterized by amyloid deposition rather than spongiform change in the affected brain tissue [4]. The genotype in codon 129 of the *PRNP* gene and the type of PrP^Sc^ are considered to be the main determinants influencing clinicopathological variability [2].

Sporadic CJD (sCJD) is characterized by widespread brain deposition of PrP^Sc^ leading to spongiform change, microglial activation, synaptic and neuronal loss, and astrocytic gliosis of variable severity and regional distribution; PrP-amyloid plaques occur in about 10% of cases [5]. The clinical picture is characterized by various combinations of rapidly progressing dementia, visual or cerebellar disturbance, extrapyramidal or pyramidal dysfunction, myoclonus and in later stages, akinetic mutism. According to the current classification, six main clinicopathological phenotypes correlating with genotypes (methionine, M or valin,V) at codon 129 of the *PRNP* gene and with two forms of PrP^Sc^ (types 1 and 2) on Western blot are distinguished within sCJD [3]. Specifically, each phenotype variant results from a specific codon 129 genotype/PrP^Sc^ combination, which in fact predetermines the morphology (fine vs. coarse) and anatomical distribution (neocortex, basal ganglia, thalamus, cerebellum) of PrP^Sc^ immunodeposities and thus the clinical phenotype and duration of the disease [2]. In addition to prion protein, several cases of concomitant neurodegenerative proteinopathy have been reported in sCJD, including AD, LBD, AGD or PSP. In some cases, combinations of AD+LBD+AGD or PSP+AD+AGD have been found [6]. Interestingly, the existence of more than one major neurodegenerative -associated protein was significantly associated with better survival in sCJD [6].

Although some forms of human prion diseases, specifically GSS, have long been classified as so-called secondary tauopathies [4], concomitant tauopathy in cases of sCJD is rather rare [7]. Primary age related tauopathy (PART) and age-related tau astrogliopathy (ARTAG) are considered age-related tauopathies. PART designates cases with NFTs in the medial temporal lobe without significant concomitant Aβ depositions [8]. ARTAG is characterized by astrocytic tau immunoreactivity such as thorn-shaped astrocytes (TSA) or granular–fuzzy astrocytes (GFA) detectable in various anatomical regions in subpial, subependymal, perivascular and grey or white matter predominant localizations [9]. Both PART and ARTAG are so far only pathological units, described mainly in ageing brains, mostly in cases older than 75 years. They have also been reported in a few cases in association with sCJD, but also in individuals over 70 years of age [7]. It has been discussed whether these are independent processes, a coincidental co-occurrence, or whether there may be some association. There have been speculations about the possibility of accelerating the course of pre-existing tauopathy or the possibility of accelerating the ageing process in the CJD brains [7].

Here we present the clinical course and neuropathological findings of a patient with CJD in whom the above-mentioned tauopathies PART and ARTAG, considered to be typical for older age, were found as early as 58 years of age. Further we discuss the knowledge gained so far regarding the relationship between concomitant tauopathy and various forms of CJD, including PART and ARTAG in sCJD. According to the available information, the presented case represents an unusually early occurrence of age-associated tauopathies not only in relation to sCJD, but also in general.

## Results

### Neuropathological findings

**Figure 1.**
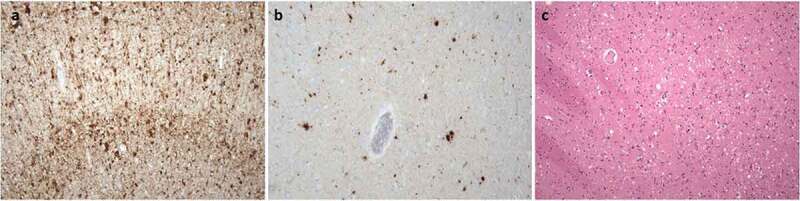
Neuropathological finding showing changes corresponding to Creutzfeldt-Jakob disease: (a) Diffuse synaptic positivity and enhanced perivacuolar ‘patchy’ positivity and ‘plaque-like’ positive structures in cerebral cortex, stained with monoclonal antibody (12F10) against prion protein, original magnification 200. (b-c) Diffuse synaptic positivity and enhanced perivacuolar ‘patchy’ positivity and ‘plaque-like’ positive structures in striatum, stained with monoclonal antibody (6H4) against prion protein, original magnification 200x and H&E staining, original magnification 100x

**Figure 2.**
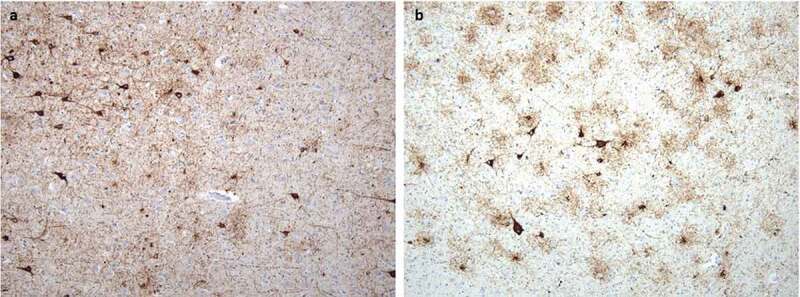
Neuropathological finding corresponding to concomitant tauopathies: (a) Deposits of hyperphosphorylated tau protein in the amygdala corresponding to primary age-related tauopathy (PART); staining with monoclonal antibody against hyperphosphorylated tau (AT8), original magnification 100x. (b) Deposits of hyperphosphorylated tau protein in the amygdala corresponding to age-related tau astrogliopathy (ARTAG); staining with monoclonal antibody against hyperphosphorylated tau (AT8), original magnification 100x

Neuropathological, immunohistochemical, neuro-immunological, and molecular genetic examinations definitively confirmed the final diagnosis of sporadic Creutzfeldt–Jakob disease, MM2 subtype. Spongiform dystrophy and numeric atrophy of neuronal structures accompanied by reactive astrogliosis were explored in cortical and subcortical structures. Immunohistochemistry, which was positive in two different monoclonal antibodies against the prion protein (clones 6H4 and 12F10), showed diffuse synaptic positivity, moreover, accented perivacuolar, so-called ‘patchy’ positivity and ‘plaque-like’ positive structures predominantly in basal ganglia were also seen locally (Figure 1). As an accessory neuropathological finding, deposits of the hyper-phosphorylated form of tau protein (clone AT8) of different types were found in the hippocampal formation, temporal cortex, striatum, amygdala and mecencephalic structures, which precise classification is difficult, however partially meet the criteria for combined ageing-related tauopathy sharing picture of primary age related tauopathy (PART) and age-related tau astrogliopathy (ARTAG) (Figure 2).

## Discussion

Concomitant neurodegenerative tauopathy is a relatively constant finding in inherited PrP amyloidosis, especially GSS [4]. The presence of tauopathy in CJD has been documented relatively recently. The small neuritic profiles are regarded as the most frequent type of tau immunoreactivity in CJD brains [7,10]. Tau protein deposits in CJD are mostly present as tiny rod or stump-like punctate inclusions in the neuropil or as thin neuritic threads [7,10,11]. The morphology and density of hyperphosphorylated tau deposits correspond to the pattern of PrP^Sc^ deposition and the morphology of PrP^Sc^ aggregates [11]. Higher density of PrP^Sc^ deposits and small plaque-like deposits are associated with stronger tau protein deposition, while perineuronal PrP^Sc^ aggregates were associated with lower tau aggregate densities. So, for a long time it was thought that only prion disease-forming plaques are able to generate phospho-tau deposits, whereas forms with PrP synaptic deposits may not be able to do so [12]. Later, in a study comparing histopathological findings in the brain tissue of cases with sCJD and cases with inherited CJD and vCJD, the presence of hyperphosphorylated tau deposits was also demonstrated in sCJD with synaptic or pericellular PrP [11]. These correlated with small tau granules showing homogeneous distribution, while coarse granular PrP^Sc^ colocalized with larger and well discernible tau granules [11]. Another finding of this study was that tau deposition in sCJD are not limited to the hippocampus and neocortical structures, but also involve the granular, molecular and Purkinje cell layers of the cerebellum [11]. When evaluating the degree of pathological tau protein involvement, tauopathy was much more pronounced in sCJD subtypes associated with strain V2 (i.e., subtypes VV2 and MV2K) in which accumulated PrP^Sc^ is present in the form of kuru-like plaques and plaque-like deposits [11]. This fact together with the high prevalence of tauopathy in PrP-amyloidosis suggests a possible relationship between structural properties of PrP^Sc^ aggregates and pathological conformation of tau protein.

Much less is known about the presence of NFT pathology in sCJD. NFT pathology is one of the main features of ageing brain and the hierarchy of its development is well documented in the frame of pathology related to Alzheimer disease. This process that starts in the entorhinal cortex and spreads over the limbic system and finally extends into neocortical regions has been characterized in detail and formalized in a staging system by Braak and Braak [13,14]. NFT pathology is a very common finding in PrP amyloidosis especially in GGS.

A recent and essentially the only study systematically investigating the presence of NFT tauopathy has been performed by Kovacs et al. [7]. They reported the presence of NFT tauopathy in the medial temporal lobe compatible with primary age-related tauopathy (PART) in almost 70% cases. This hippocampal NFT pathology was not compatible with Braak staging in up to one third of cases. This difference compared to AD cases was due to distinct involvement of the entorhinal cortex together with the increased tau deposition in the dentate gyrus and CA4 subregion. Furthermore, a more widespread pathology was found in this study. This included AGD-compatible changes in several cases including one with subcortical neurofibrillary tangles as in early forms of PSP and a prominent astroglial tau pathology, including grainy/fuzzy astrocytes (GFA) in grey matter regions, subpial, subependymal, perivascular and white matter thorn-shaped astrocytes (TSA) compatible with the ARTAG type pathology [7].

From the above mentioned, it is clear that the presence of tau pathology in CJD brains is not uncommon. However, the interrelationship between prion protein and tau protein remains a matter of debate in these cases. Is it an independent process or a random coincidence or a common pathogenetic process plays a role here? Molecular interactions between PrP and tau protein indicating a potential role for tau in the biological function of PrP and the pathogenesis of CJD have been demonstrated in animal models [15]. Structural properties of PrP^Sc^ aggregates, in particular amyloid, which contributes to the phosphorylation of tau protein, are likely to play a role, which is reflected in the frequent occurrence of tau pathology in PrP amyloidosis [11]. An unanswered question remains the relationship between prion protein and hippocampal NFT tau pathology without amyloid deposits (PART and ARTAG pathology) in sCJD. It is important to consider that prion disease may occur in pre-existing neurodegenerative disease and cause a modification in the acceleration of the clinical course. Or since many cases are under the age of 75, there is speculation that the ageing process in CJD brains may accelerate [7]. The real question, however, is whether we can assume the presence of pre-existing PART and ARTAG pathologies, typical age-related proteinopathies, as early as 58 years of age.

## Patient and methods

### Clinical summary

The patient developed the first problems between the ages of 55 and 60. These included worsening of fine motor skills and gradual progression of cognitive impairment, which were observed by the patient’s family. Three months after the onset of the problem, the patient underwent a magnetic resonance imaging (MRI) of the brain at a regional hospital, which showed only minor non-specific changes in white matter bilaterally. Approximately 8 months after the onset of the problems, the patient was admitted to hospital. The family members stated that the patient is unable to take care of the household, needs assistance in performing normal daily activities, including hygiene, gets lost even in a familiar environment, and sometimes the tremor of the upper limbs appears. Psychological examination revealed a cognitive deficit at the level of moderate to severe dementia. Disorientation, impairment of both short-term and long-term memory, impairment of verbal fluency and visual-spatial orientation were the predominant symptoms. Neurological examination was limited by poor patient cooperation, but in the objective neurological finding mild extrapyramidal symptoms including bradykinesia and hypokinesia along with brisk tendon reflexes and positive Babinski sign were clearly present. Furthermore, the patient suffered from significant motor restlessness with a tendency to constantly move around the department. A speech therapy examination was also performed. The finding was evaluated as a severe disintegration of the communication-cognitive processes. Electroencephalographic examination (EEG) showed a moderate to severe abnormal finding, with diffuse slowing of basal activity. The specific periodic sharp and slow wave complexes (PSWC) were not present. CSF (cerebrospinal fluid) examination revealed mild proteinorhachia along with marginally elevated level of phosphorylated tau protein and more than four-fold increased level of total tau protein. Due to the positive family history of dementia in paternal grandparents, a genetic testing involving the analysis of *MAPT, PRGN, APP, PSEN1, PSEN2* and *PRNP* genes was also performed to rule out hereditary forms of dementia. No pathogenic mutation was found in any of these genes. H1/H2 haplotype was found in the *MATP* gene. Two homozygous polymorphisms have been found in the *PRNP* gene that can modify susceptibility to sporadic CJD. These were methionine (ATG/ATG) (p.Met129)(c.385AA), dbSNP: rs179999 at codon 129 and glutamate at codon 219 (GAC/GAC) (p.Glu219)(c.655GG), dbSNP: rs1800014. MRI of the brain, in contrast to the previous examination performed 5 months earlier, showed changes in the temporo-parieto-occipital cortex bilaterally. These were hypersignal changes in the FLAIR sequences and diffusion restriction in the DWI sequences. There were no changes in the basal ganglia. During the further course of the disease, there was a rapid progression of cognitive deficit, the patient became bedridden and completely dependent on nursing care. She died due to bronchopneumonia after 14 months of the disease course.

### Clinical examination

The patient was thoroughly examined and followed up in the Tertiary Movement Disorders Center. The examination was performed according to the standard protocol used in all patients with suspected neurodegenerative disease; it included detailed clinical neurological, neuropsychological and psychiatric examination, examination of neurodegenerative markers in cerebrospinal fluid, electroencephalographic examination, magnetic resonance imaging of the brain and genetic examination. All steps of the study were approved by the local ethics committee of the University Hospital Olomouc.

### Neuropathological examination

Neuropathological examination of brain tissue was performed with the prior informed consent of the patient and her relatives. Neuropathological diagnoses, including prion protein immunoassays, were established according to standard protocols National CJD Research & Surveillance Unit. Protocol: Surveillance of CJD in the UK [16] used by the National reference laboratory for human prion diseases at the Department of Pathology and Molecular Medicine, Prague, Czech Republic. For immunohistochemical detection of other proteinopathies, 5 μm-thick sections of formalin-fixed and paraffin-embedded tissue were incubated with primary antibodies against the following antigens: α-synuclein (1:5000, Signet, Dedham, MA, USA, 4D6); tau AT8 (1:200, Pierce Biotechnology, Rockford, IL, USA, pS202/pT205); tau, 3-repeat isoform DR3 (1:500, Upstate Biotechnology, Lake Placid, NY, USA, 8E6/C11); tau, 4-repeat isoform DR4 (1:200, Upstate Biotechnology, Lake Placid, NY, USA, 1E1/A6); β-amyloid peptide (1:100, Dako, Glostrup, Denmark, 6/3D).
